# Aberrant Mitochondrial Homeostasis in the Skeletal Muscle of Sedentary Older Adults

**DOI:** 10.1371/journal.pone.0010778

**Published:** 2010-05-24

**Authors:** Adeel Safdar, Mazen J. Hamadeh, Jan J. Kaczor, Sandeep Raha, Justin deBeer, Mark A. Tarnopolsky

**Affiliations:** 1 Department of Kinesiology, McMaster University, Hamilton, Ontario, Canada; 2 Department of Pediatrics, McMaster University, Hamilton, Ontario, Canada; 3 Department of Medicine, McMaster University, Hamilton, Ontario, Canada; 4 Department of Surgery, McMaster University, Hamilton, Ontario, Canada; 5 School of Kinesiology and Health Science, York University, Toronto, Ontario, Canada; Ohio State University, United States of America

## Abstract

The role of mitochondrial dysfunction and oxidative stress has been extensively characterized in the aetiology of sarcopenia (aging-associated loss of muscle mass) and muscle wasting as a result of muscle disuse. What remains less clear is whether the decline in skeletal muscle mitochondrial oxidative capacity is purely a function of the aging process or if the sedentary lifestyle of older adult subjects has confounded previous reports. The objective of the present study was to investigate if a recreationally active lifestyle in older adults can conserve skeletal muscle strength and functionality, chronic systemic inflammation, mitochondrial biogenesis and oxidative capacity, and cellular antioxidant capacity. To that end, muscle biopsies were taken from the *vastus lateralis* of young and age-matched recreationally active older and sedentary older men and women (N = 10/group; ♀  =  ♂). We show that a physically active lifestyle is associated with the partial compensatory preservation of mitochondrial biogenesis, and cellular oxidative and antioxidant capacity in skeletal muscle of older adults. Conversely a sedentary lifestyle, associated with osteoarthritis-mediated physical inactivity, is associated with reduced mitochondrial function, dysregulation of cellular redox status and chronic systemic inflammation that renders the skeletal muscle intracellular environment prone to reactive oxygen species-mediated toxicity. We propose that an active lifestyle is an important determinant of quality of life and molecular progression of aging in skeletal muscle of the elderly, and is a viable therapy for attenuating and/or reversing skeletal muscle strength declines and mitochondrial abnormalities associated with aging.

## Introduction

Individuals over the age of 69 y represent one of the fastest growing segments of the North American population [1]. Although an important public health outcome continues to be an increase in life expectancy, of even greater importance is that extended life span is accompanied by an improved capacity to function independently and a better quality of life. Aging is a multidimensional process that is influenced by genetic polymorphisms, nutrition, lifestyle and overall health status [2], [3]. One of the most striking and debilitating age-associated alterations is the progressive loss of fat-free skeletal muscle mass, a phenomenon known as sarcopenia [4]–[7]. This loss of muscle mass and strength, and the increase in body fat with aging are physiological phenomena that occur in part as a consequence of metabolic changes associated with a sedentary lifestyle in older adults [8], [9]. A chronic sedentary lifestyle can lead to “frailty,” a multidimensional geriatric syndrome of decreased resistance to stressors resulting in cumulative systemic declines [10]–[13]. Clinical trials have shown that sedentary older adults significantly improved their physical performance and health following a physical activity intervention [14], [15]. Longitudinal studies have shown that regular physical activity may extend life expectancy, reduce morbidity (cancer, neurological disease, etc.), and reduce physical disability in later life [2], [3], [16]–[19]. These findings suggest that preserving mobility and an active lifestyle is essential in maintaining a high quality of life in older adults.

The role of mitochondrial abnormalities and oxidative stress in the etiology of sarcopenia has been extensively characterized [20]–[27]. The “mitochondrial theory of aging” stipulates that the aging process is modulated by reactive oxygen species (ROS)-mediated toxicity leading to mitochondrial DNA (mtDNA) deletions and mutations, macromolecular oxidation, electron transport chain (ETC) dysfunction, cellular senescence and cell death [28]–[30]. Muscle from older adults show: (1) an increase in mitochondrial ETC abnormalities marked by the accumulation of cytochrome *c* oxidase negative and succinate dehydrogenase hyper-positive fibres, (2) an increase in markers of oxidative stress, (3) accumulation of somatic mtDNA mutations, and (4) a transcriptome “signature” indicative of mitochondrial dysfunction [21], [22], [31]–[33]. Despite a strong relationship between aging and oxidative damage, the literature on the effect of aging on skeletal muscle ETC function remains equivocal in humans. Many studies have demonstrated a significant age-related reduction of mitochondrial ETC complex enzymes in human skeletal muscle [34]–[37], while others have not observed such changes [33], [38]–[41]. Barrientos and colleagues (1996) suggested that the reported age-related reduction in ETC function (reduced mitochondrial complex I, II, III, and IV activity) is not related to the aging process *per se*, but rather due to other confounding factors, including physical inactivity [38]. Our group has also reported normal mitochondrial ETC function in the skeletal muscle of recreationally active elderly individuals (72±2 y) despite an increase in markers of oxidative damage (e.g., protein carbonyls and 8-hydroxy 2-deoxyguanosine [8-OHdG]) *vs.* healthy young individuals (22±3 y) [33]. Hence, the relationship between mitochondrial ETC dysfunction, oxidative stress and sarcopenia remain an important and unresolved issue in aging research that is likely influenced by physical activity status.

The principal aim of this study was to investigate the equivocal findings regarding mitochondrial oxidative capacity in human skeletal muscle aging using two age-matched older adult populations, a recreationally active old (AO) and sedentary frail old (SO), who differed primarily in their physical activity status. In addition, we also delineated if a physically active lifestyle can: (1) attenuate the loss of skeletal muscle strength and functional capacity, (2) reduce systemic inflammation, (3) conserve mitochondrial biogenesis and complex IV activity (indicative of mitochondrial oxidative capacity), and (4) maintains cellular redox status via maintenance of mitochondrial and cytosolic superoxide dismutase (first line of defence against ROS). We hypothesized that the skeletal muscle strength, systemic inflammation, mitochondrial oxidative capacity, and antioxidant response would be relatively better preserved in the physically active, but not sedentary frail, elderly *vs.* the young.

## Methods

### Study Participants and Experimental Protocol

We recruited recreationally active university young students, recreationally active old (AO), and sedentary old (SO) men and women from Hamilton area for this cross-sectional study (Table 1). Both young and active old subjects carried out activities of daily living (walking, grocery shopping, gardening, etc.) and participated in modest recreational activities (golfing, gardening, tennis and/or cycling) three or more times a week but were not competitive athletes, and were healthy. Consequently, the young and AO subjects had similar volumes and intensities of physical activity. The SO subjects were patients with a primary diagnosis of knee joint osteoarthritis that rendered them with a sedentary lifestyle, but were otherwise fairly healthy. The SO subjects were recruited through the total knee-joint arthroplasty program at the Hamilton Health Sciences Corporation. The inclusion criteria for the SO group included: evidence of severe knee joint osteoarthritis by radiography, age 50–75 y, and no previous joint arthroplasty in the knee to be operated upon (and biopsied, see below). The exclusion criteria for all subject groups in the study included: evidence of coronary artery disease, congestive heart failure, renal failure (creatinine >120 µmol/L; potassium >5.00 mmol/L), diabetes requiring insulin or a glyburide dosage of 5 mg or more, previous stroke with motor loss, rheumatoid or other known inflammatory arthritis, uncontrolled hypertension or hypertension requiring more than monopharmacotherapy, inability to give consent because of cognitive difficulties, chronic obstructive pulmonary disease (forced vital capacity or forced expiratory volume in 1 second <85% of age-predicted mean value, or requiring any medication other than an inhaler as needed), and smoking. The elderly women (both AO and SO) were post-menopausal and were not taking hormone replacement therapy, and the young women were not taking oral contraceptives. All subjects provided written consent prior to their participation. The study was approved by the Hamilton Health Sciences Human Research Ethics Board and conformed to the guidelines outlined in the Declaration of Helsinki.

**Table 1:** Study subjects' characteristics.

	Young	AO	SO
Sample size (N)	10^‡^	10^‡^	10^‡^
Age (y)	22***±***2	70***±***5	63***±***10
Weight (kg)	70***±***17	74***±***15	80***±***15
Height (cm)	175***±***11	164***±***7	169***±***10
Fat-free mass (kg)	55***±***10	55***±***12	45***±***4*
Body fat (percentage)	19***±***6	29***±***11^†^	45***±***9*
BMI (kg/m^2^)	23***±***2	28***±***3^†^	28***±***5^†^
Maximal isometric torque (N.m)	220***±61***	***119±34*** ^†^	***53±24*** ^*^
Skeletal muscle CRP (ng.mg of protein^−1^)	0.54***±0.34***	***1.25±0.84*** ^†^	2.27±0.74*

Values are means ± SD. ^‡^♀  =  ♂; ^*^ significantly different from young and AO (P<0.05); ^†^significantly different from young (P<0.05).

### Body Composition and Strength Testing

We measured total body weight and height of the subjects to the nearest 0.1 kg and 0.5 cm, respectively, using a calibrated electronic scale (Health-O-Meter Pro Series Electronic Scale, Bridgeview, IL). We also assessed body composition was using bioelectric impedance (RJL Systems, Clinton Twp, MI) and maximal isometric torque of subject's right leg using a dynamometer (Biodex System 3, Biodex Medical Systems, Shirley, NY) as previously described by our group [42], [43]. For body composition assessment of SO subjects, the electrodes were placed on the non-arthroplasty leg and the ipsilateral arm. To determine maximal isometric contraction, subjects were positioned into the machine with the knee flexed at 90° and performed three maximal 5-second voluntary contractions with 30-second rest between each trial.

### Functional Testing

We assessed functional capacity of elderly subjects (AO and SO), as an indirect marker of their cardiovascular fitness and hence physical activity status, via a timed stair-climb test and a walk test using a stopwatch that recorded times to an accuracy of 1/100 of a second. The 30-feet walk test comprised of subjects walking as fast and safely as possible for a 30-feet distance that was marked off with clear start and stop points. The stair-climb test included subjects climbing four stairs, starting with both feet on the bottom platform and ascending one step at a time, using a handrail only if insecure.

### Muscle Biopsy

All subjects were instructed to abstain from physical activity for 48-h prior to the muscle biopsy. Young and AO subjects arrived at McMaster University Hospital (Hamilton, Ontario, Canada) between 0800 h and 0930 h in the post-absorptive state following an overnight fast. We collected a muscle biopsy of the *vastus lateralis* of the dominant leg, 10 cm proximal or distal to the knee joint using a modified Bergström needle (5 mm diameter) with suction modification [44]. After quickly dissecting the biopsied muscle tissue of fat and connective tissue, we placed ∼100 mg of wet muscle in RNase-free cryoviles, immediately snap froze it in liquid nitrogen, and stored at −86°C until analysis. SO subjects were biopsied from the *vastus lateralis* at the time of their total knee joint arthroplasty procedure at McMaster University Hospital (Hamilton, Ontario, Canada) immediately after the first incision and without vascular occlusion, 10 min after induction. Biopsies from SO subjects were collected by a single surgeon and the wet muscle tissue was stored as aforementioned.

### Skeletal Muscle Tissue Homogenization

The total protein was extracted from the frozen skeletal muscle samples as described previously in detail by our group [45]. Briefly, ∼30 mg of skeletal muscle was homogenized on ice in a 2 mL Wheaton glass homogenizer (Fisher Scientific, Ottawa, ON) with 25 volumes of phosphate homogenization buffer [50 mM KH_2_PO_4_, 5 mM EDTA, 0.5 mM DTT, 1.15% KCl] supplemented with a Complete Mini, ETDA-free protease inhibitor cocktail tablet and a phosphatase inhibitor cocktail tablet (PhosSTOP, Roche Applied Science, Mannhein, Germany) per 10 mL of buffer. The lysate was centrifuged at 600 *g* for 15 min at 4°C to pellet cellular debris. The supernatant was aliquoted, snap frozen in liquid nitrogen and stored at −86°C until further analysis. The Lowry assay was used to quantify the total protein content of samples.

### Marker of Systemic Inflammation

Muscle lysates were also analyzed for C-reactive protein (CRP; Alpha Diagnostic International, San Antonio, TX) content using an enzyme immunoassay, as per manufacturer instructions. All samples, standards, and controls were run in duplicate and results were expressed as ng•mg protein^−1^ for CRP.

### Immunoblotting

Proteins were resolved on 7.5, 10 or 12.5% SDS-PAGE gels depending on the molecular weight of the protein of interest. The gels were transferred onto Hybond® ECL nitrocellulose membranes (Amersham, Piscataway, NJ), and immunoblotted using the following commercially available primary antibodies: anti-COX subunit I (cytochrome *c* oxidase - subunit I; MS404), anti-COX subunit II (cytochrome *c* oxidase - subunit II; MS405) and anti-COX subunit IV (cytochrome *c* oxidase - subunit IV; MS408, MitoSciences, Eugene, OR); anti-Cu/Zn-SOD (copper/zinc superoxide dismutase; ab16831), anti-Mn-SOD (manganese superoxide dismutase; ab13533) and anti-nNOS (neuronal nitric oxide synthase; ab63602, Abcam, Cambridge, MA); anti-PGC-1α (2178, Cell Signaling Technology, Denver, MA). The anti-CS (citrate synthase) antibody was a generous gift by Dr. Brian Robinson (The Hospital for Sick Children, Toronto, ON). Anti-actin (Novus Biologicals, Littleton, CO) was used as a house-keeping loading control and to normalize the expression of proteins of interest. All antibodies were used at 1∶1000 dilution, except for anti-COX subunit II (1∶4000 dilution), anti-CS (1∶2000 dilution) and anti-actin (1∶10,000 dilution). Membranes were then incubated with the appropriate anti-mouse or anti-rabbit (depending on the primary antibody source) horse radish peroxidase-linked secondary antibody (1∶5000 dilution) and visualized by enhanced chemiluminescence detection reagent (Amersham, Piscataway, NJ). Relative intensities of the protein bands were digitally quantified by using NIH ImageJ, version 1.37, analysis software (Scion Image, NIH).

### Citrate Synthase and Cytochrome *c* Oxidase Activity

Citrate synthase (EC 2.3.3.1) activity was determined by measuring the formation of thionitrobenzoate anion, as previously described by our group [33]. Briefly, 15 µL of muscle homogenate was added to 810 µL buffer (0.1M Tris–HCl buffer, pH 8.0) along with 10 µL of acetyl CoA (7.5 mM in 0.1M Tris-HCL buffer, pH 8.0) and 100 µL of 0.1 mM dithionitrobenzoic acid. The reaction was started by adding 50 µL of 9.0 mM oxaloacetate. Absorbance was recorded at 412 nm every 30 s for 3 min at 37°C. CS activity was expressed in nmol.min^−1^.mg of protein^−1^. Mitochondrial electron transport chain cytochrome *c* oxidase (COX; EC 1.9.3.1) activity (indicative of mitochondrial oxidative capacity) was determined by measuring the rate of oxidation of reduced cytochrome *c*, as previously described by our group [33]. Briefly, stock cytochrome *c* (oxidized) was reduced by sodium ascorbate in 0.05 mM potassium phosphate buffer (KH_2_PO_4_, pH 7.4). Fifteen microliters of muscle homogenate were added to 955 µl of 0.05 mM potassium phosphate buffer, and 15 µL of reduced cytochrome *c*. Absorbance was recorded at 550 nm every 30 s for 3 min at 37°C. All samples were analyzed in duplicate on a spectrophotometer (Cary Bio-300, Varion, Inc., Palo Alto, CA). The intra-assay coefficient of variation for all samples was less than 4%.

### Antioxidant Enzyme Activity

Muscle total superoxide dismutase (Mn-SOD and Cu/Zn-SOD; EC 1.15.1.1) activity was determined in muscle lysate by measuring the kinetic consumption of superoxide radical (O_2_^-^) by SOD in a competitive reaction with cytochrome *c*, as previously described by our group [33]. Absorption was recorded at 550 nm and was observed every 15 s for 2 min at 37°C. One unit (U) of SOD activity was defined as the amount of enzyme that caused a 50% inhibition of the reduction of cytochrome *c*. Total SOD activity was expressed in U•mg of protein^−1^. In a separate cuvette, the same sample was analyzed under identical conditions in the presence of 0.2 M KCN (pH 8.5–9.5), a potent inhibitor of cytosolic Cu/Zn-SOD [46], for determination of mitochondrial Mn-SOD activity. Cu/Zn-SOD activity was approximated by subtracting Mn-SOD activity from total SOD activity. Both Mn-SOD and Cu/Zn-SOD activity were expressed in U•mg protein^−1^. All samples were analyzed in duplicate on a spectrophotometer.

### Mn-SOD Immunoprecipitation and Nitration Analyses

Muscle lysate (100 µg) was pre-cleared in 25% v/v pre-clearing matrix F (sc-45057; Santa Cruz Biotechnology, Santa Cruz, CA) overnight at 4°C. The supernatant was then incubated with 10 µg of polyclonal anti-Mn-SOD antibody (ab13533; Abcam, Cambridge, MA) and ExactaCruz™ F matrix (sc-45043; Santa Cruz Biotechnology, Santa Cruz, CA) complex with mixing by end-over-end inversion overnight at 4°C. The matrix was centrifuged at 16,000 *g* for 30 sec, and the pellet matrix-immune complex precipitate was washed with 500 µL of PBS buffer (5 times). The washed matrix-immune complex was re-suspended in Laemmli sample buffer supplemented with 5% β-mercaptoethanol, and heated at 95°C for 5 min. After centrifugation at 16,000 *g* for 1 min, samples were immediately loaded and resolved on reducing 12.5% SDS-PAGE gels. The nitrocellulose membranes were blotted for Mn-SOD to confirm immunoprecipitation. Repeat addition of anti-Mn-SOD antibody - ExactaCruz™ F matrix showed no further immunoprecipitated protein, indicating that the procedure is quantitative. To quantify Mn-SOD nitration, Mn-SOD immune complex were resolved on reducing 12.5% SDS-PAGE gels, followed by immunoblotting with primary anti-nitrotyrosine polyclonal antibody (06-284, 1∶1000 dilution; Millipore™, Billerica, MA) and secondary anti-rabbit horse radish peroxidise antibody (1∶5000 dilution). Nitration of Mn-SOD was digitally quantified as aforementioned.

### Statistical Analysis

Anthropometric measurements, maximal isometric torque, protein content (normalized to actin), enzyme activity measurements and CRP content between the groups were analyzed using one-way analysis of variance (ANOVA) using Statistica 5.0 software (Statsoft, Tulsa, OK.). Functional capacity measures of elderly subjects (AO and SO) were analyzed using an unpaired Student's *t*-test using Statistica 5.0 software (Statsoft, Tulsa, OK.) For all analyses, a two-tailed test was employed. We used Tukey's HSD test post-hoc to identify individual differences when statistical significance was observed. Statistical significance was established at a P≤0.05. Data are presented as mean ± standard deviation (SD).

## Results

### Physical inactivity mediates systemic inflammation, and loss of skeletal muscle mass, strength, and functional capacity

**Figure 1:**
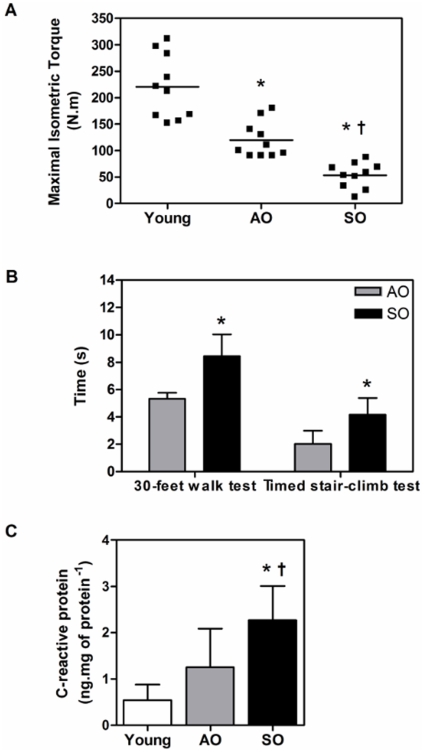
A sedentary lifestyle exacerbates functional decline and systemic inflammation in the elderly. (A) Maximal isometric torque (N.m) in young, AO, and SO subjects. Each point represents an individual who underwent strength testing as described in the methods. (B) Walk test and timed stair-climb test in AO and SO subjects as described in the methods. (C) CRP levels (ng.mg of protein^−1^) in the *vastus lateralis* of young, AO, and SO subjects (N = 10/group; ♀  =  ♂). Asterisk denotes significant changes *vs.* young, and dagger denotes significant changes *vs.* AO (P≤0.05).

Fat-free mass was significantly lower while percent body fat was significantly higher in SO group relative to both the young and AO groups (P<0.05) (Table 1). Interestingly, there was no difference in body mass index between SO and AO groups (Table 1). The latter findings are of importance by emphasizing that accurate characterization of older adult anthropometric characteristics requires measurements of body fat and fat-free mass and that BMI is not a sensitive parameter for sarcopenia. We also measured maximal isometric torque as a marker of muscle strength and found that the force generated by the SO individuals was significantly lower in comparison to both the young and AO groups (76% and 56%, respectively; P<0.01) (Figure 1A). Also, the AO group generated significantly less torque than the young group (46%; P<0.01). Similarly, SO subjects had reduced functional capacity as indicated by walk test and stair-climb test compared to AO group (Figure 1B). Taken together, these results suggest that an active lifestyle attenuates age-associated losses of muscle mass and strength.

Studies have shown that basal levels of pro-inflammatory cytokines, such as interleukin 6 (IL-6) and C-reactive protein, are lower in physically active individuals [47], [48]. CRP, a marker of chronic systemic inflammation negatively associated with physical activity, was significantly elevated in the skeletal muscle of the SO group relative to both the young and AO groups (297% and 82%, respectively; P<0.05) (Figure 1C). This shows that muscle inactivity in SO promotes low-grade chronic inflammation in skeletal muscle of elderly.

### Mitochondrial biogenesis, abundance and COX are compromised in sedentary older adults

**Figure 2:**
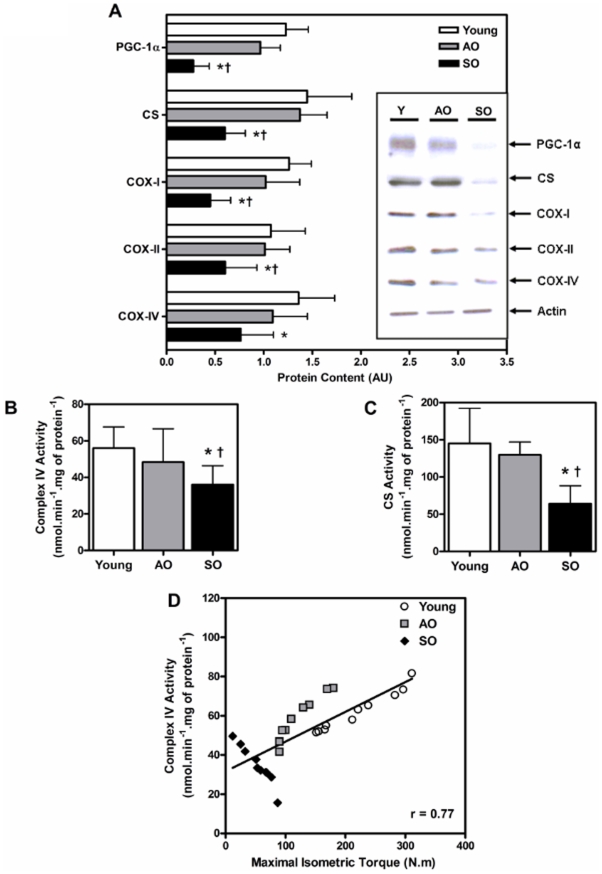
Mitochondrial biogenesis and COX activity are impaired in frail old. (A) PGC-1α, CS, COX subunits- I, II, and IV, and phospho-GSK3β (Ser^9^) protein content, (B) citrate synthase and (C) mitochondrial complex IV activity (nmol.min^−1^.mg of protein^−1^) in the *vastus lateralis* of young, AO, and SO subjects (N = 10/group; ♀  =  ♂). (D) Mitochondrial complex IV activity (nmol.min^−1^.mg of protein^−1^) positively correlates (r = 0.77) with maximal isometric torque (N.m). Asterisk denotes significant changes *vs.* young, dagger denotes significant changes *vs.* AO, and double dagger denotes significant changes *vs.* both young and AO (P≤0.05).

The SO group had significantly lower citrate synthase, PGC-1α, and COX subunits- I and II (mtDNA-encoded) protein content than both the young (58%, 78%, 64%, and 44%, respectively; P<0.003) and AO (56%, 72%, 56%, and 30%, respectively; P<0.03) groups (Figure 2A). However, the protein content of COX subunit-IV (nuclear DNA-encoded) in the SO group was 44% lower than the young group only (P = 0.01) (Figure 2A). In addition, the SO group had 63% higher levels of phosphorylated GSK3β (Ser^9^) relative to the AO group (P = 0.040; Figure 2A). Both the mitochondrial citrate synthase (Figure 2B) and COX (Figure 2C) activity were significantly lower in the SO group in comparison with both the young (56% and 36%, respectively; P<0.01) and AO (42% and 25%, respectively; P<0.01) groups. Importantly, there were no significant differences in any of these measures between the young and AO groups. This suggests that mitochondrial biogenesis and ETC function in the skeletal muscle remain relatively unchanged by aging in physically active individuals, and that hypodynamia negatively affects both the mitochondrial abundance and COX activity, indicative of reduced mitochondrial oxidative capacity. This result likely explains the controversy regarding the skeletal muscle mitochondrial ETC enzyme activity with aging; namely, that differences in mitochondrial capacity between young and older adults is likely a function of both chronological age as well as biological age (the latter due primarily to physical activity levels) [33]–[38], [40], [41]. These findings emphasize the critical importance of defining the activity level of participants when comparing young and old and/or evaluating the potency of an intervention in attenuating or reversing aging-associated pathologies. Interestingly, we observed a significant correlation between COX activity and maximal isometric torque (R = 0.77, P<0.001) looking across all the participants in the study (Figure 2D). This clearly demonstrates that muscle strength is directly associated with the muscle mitochondrial oxidative capacity.

### Mitochondrial Mn-SOD activity is preserved in recreationally active, but not sedentary, older adults

**Figure 3:**
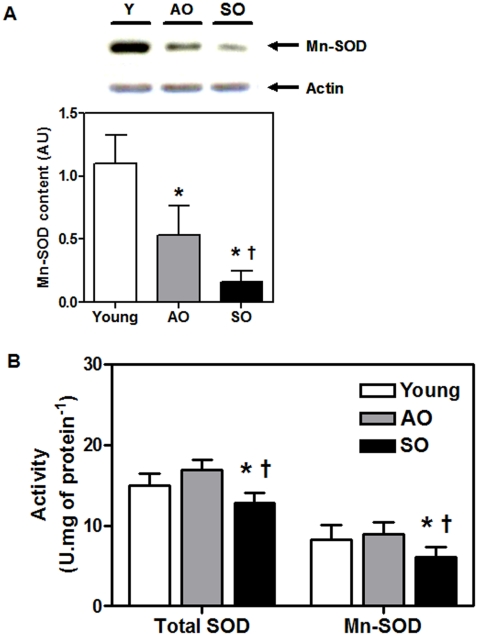
Mitochondrial Mn-SOD activity is reduced in frail old. (A) Mn-SOD protein content and (B) total- and Mn- SOD activity (U.mg of protein^−1^) in the *vastus lateralis* of young, AO, and SO subjects (N = 10/group; ♀  =  ♂). Asterisk denotes significant changes *vs.* young, and dagger denotes significant changes *vs.* AO (P≤0.05).

During the aging process, the rate of superoxide radical (O^-.^_2_) generation increases [49]. Both cytosolic and mitochondrial superoxide dismutases function as a first line of defence against oxidative stress-mediated by O^-.^_2_ [50]. The protein content of mitochondrial Mn-SOD was significantly lower in the SO group relative to both the young and AO groups (85% and 69%, respectively; P<0.001) (Figure 3A). The total-SOD and Mn-SOD activity were significantly lower in the SO group compared with the young (14% and 26%, respectively; P<0.03) and AO (24% and 32%, respectively; P<0.03) groups (Figure 3B). Surprisingly, no significant differences in both the total-SOD and Mn-SOD activity were detected between the AO and young groups (Figure 3B); even though the AO group has 55% lower Mn-SOD protein content when compared to the young (P<0.001) (Figure 3A). There was no difference in cytosolic Cu/Zn-SOD protein content and enzyme activity between the three groups (data not shown). Since aging in skeletal muscle is widely related to increases in O^-.^_2_ production via mitochondrial ETC, we suggest that physical activity maintains mitochondrial Mn-SOD activity in AO individuals at levels observed in much younger individuals despite significant reductions in the Mn-SOD protein content.

### Mn-SOD tyrosine residue is nitrated in the skeletal muscle of desentary older adults

**Figure 4:**
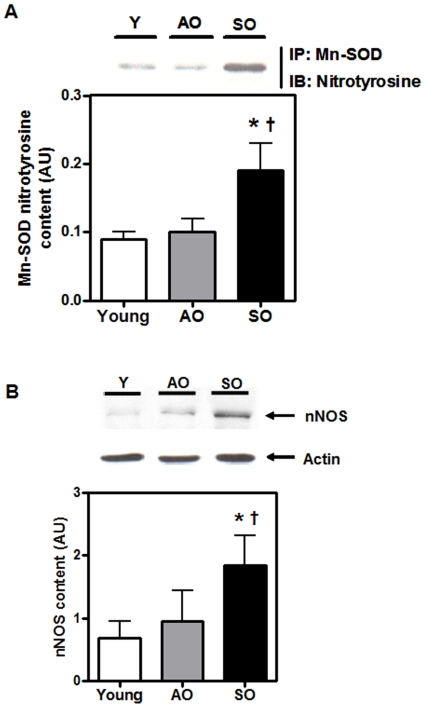
Reduced Mn-SOD activity is due to tyrosine nitration in frail old. (A) Mn-SOD was immunoprecipitated (IP) followed by immunoblotting (IB) for Mn-SOD nitrotyrosine content and (B) nNOS protein content in the *vastus lateralis* of young, AO, and SO subjects (N = 10/group; ♀  =  ♂). Asterisk denotes significant changes *vs.* young, and dagger denotes significant changes *vs.* AO (P≤0.05).

In addition to ROS, an increase in cellular peroxynitrite (ONOO^-^) radical-mediated damage has been implicated in aging-associated pathologies [51]–[55]. ONOO^-^ radical is a potent nitrating and oxidizing agent which is formed by a rapid reaction of nitric oxide (NO) with O^-.^_2_ anion. Mn-SOD is susceptible to rapid inactivation by ONOO–mediated nitration of critical active-site tyrosine residue [50]. To determine whether nitration was related to the decline of Mn-SOD activity in the SO group, Mn-SOD nitration was evaluated following immuno-precipitation of Mn-SOD. Nitrotyrosine content of immunoprecipitated Mn-SOD was significantly higher in the SO group as compared to both the young and AO groups (64% for both; P<0.001) (Figure 4A). An increase in peroxynitrite could result from increased cellular NO following an increase in inducible NOS (iNOS) expression or overactivation of endothelial NOS (eNOS) and neuronal NOS (nNOS) [50]. Thus, the protein content of nNOS was evaluated and found to be significantly higher in the SO group relative to both the AO and young groups (171% and 92%, respectively; P<0.01) (Figure 4B). Together, these results suggest that physical activity preserves Mn-SOD activity in AO individuals, likely due to attenuation of age-associated nitration of Mn-SOD. Taken together, the results suggest that hypodynamia-mediated dysregulation of antioxidant cascade makes the intracellular environment more conducive to pro-oxidant production, resulting in a cellular “redox crisis”.

## Discussion

In this study, we have shown that a sedentary lifestyle, associated with osteoarthritis mediated hypodynamia, promoted loss of muscle strength, weakness, and reductions in functional capacity, fat-free mass, and mitochondrial oxidative capacity concomitant with “low-grade” chronic inflammation in the skeletal muscle of older adults. However in recreationally active older adults, mitochondrial biogenesis and ETC function are relatively preserved in skeletal muscle. The mitochondrial antioxidant enzyme milieu of active older adults provides an intra-organellar environment conducive to O^-.^_2_ dismutation via maintenance of their Mn-SOD activity at levels similar to those found in young subjects, despite an aging-associated decline in Mn-SOD content in skeletal muscle. Conversely, the skeletal muscle of sedentary older adults fails to modulate the intracellular environment which is thus prone to ROS-mediated toxicity and aberrant redox homeostasis.

The effects of aging on skeletal muscle are invariantly characterized by a progressive loss of strength and power, muscle mass, gait velocity, endurance, and, as result, overall performance [56]–[60]. In addition to muscle loss with aging, frailty manifests as a multi-system pathology resulting in systemic weakness with low muscle strength, fatigability, exhaustion, and exercise intolerance in 20–30% of older adults [9], [61]. In this study, SO subjects had low levels of physical activity secondary to osteoarthritis, a pathology manifested solely due to daily mechanical stress-mediated wear and tear over the lifespan of an individual [62], [63]. Osteoarthritis should not be confused with rheumatoid arthritis (an autoimmune disease with joint inflammation) since inflammation is not a conspicuous feature of the disease [62]. Patients with osteoarthritis have reduced quality of life and experience a sedentary lifestyle due to pain associated with the disease [63]. Wilkie et al. (2007) also reported that knee pain severity was strongly associated with restricted mobility of subjects, further showing that joint pain (as a result of knee OA) will render patients immobile [64]. Hence, age- and gender- matched SO subjects provide an excellent sedentary control group for physically active AO subjects to study the relationship between a physically active lifestyle and skeletal muscle maintenance and bioenergetic homeostasis in the elderly. Recently, Ruiz and colleagues (2008) reported that muscular strength is inversely and independently associated with all cause mortality in men [65]. Here we demonstrate that even though older adults show an age-associated reduction in skeletal muscle maximal isometric torque relative to young adults, the loss of muscle strength in AO is dramatically attenuated in comparison to age-matched SO subjects (Figure 1A). AO subjects had higher functional capacity compared to SO subjects (Figure 1B), an indirect indication of better quality of life and functional independence which is a function of cardiorespiratory fitness and physical activity status (Mailey et al., 2010 BMC Public Health). This is consistent with our previous work where six months of resistance training resulted in significant gains in fat-free muscle mass, muscle strength and endurance, and functional capacity in older adults [45], [66]. The rate of decline in maximal aerobic capacity (VO_2max_) with age is attenuated in adults who perform regular aerobic exercise and are physically active [67], [68]. Similarly, a regular exercise regimen, physical therapy and lifestyle modification are therapeutic in alleviating pain, reducing the progression of disease and improving overall quality of life in patients with osteoarthritis [69], [70]. Better adherence to recommended home exercises as well as being more physically active improves the long-term effectiveness of exercise therapy in patients with osteoarthritis of the knee [63], [69]–[71]. Taken together, the aforementioned results imply that physical activity is an effective counter-measure to sarcopenia, preserving skeletal muscle function and independence in the elderly.

Frailty and disability in older adult muscle is also associated with increased production of systemic pro-inflammatory cytokines [72], [73]. Higher serum levels of markers of inflammation in the elderly are associated with several age-associated co-morbidities including dementia, Parkinson's disease, atherosclerosis, type 2 diabetes, sarcopenia, functional disability, and are strong independent risk factors of morbidity and mortality in the elderly [74], [75]. CRP is an acute phase reactant and is a marker of systemic inflammation that is under direct IL-6 transcriptional control [9]. Physical inactivity is associated with elevated serum IL-6 and CRP concentrations [47], [76]. In this study, we observed that sedentary lifestyle promotes “low-grade” chronic inflammation in skeletal muscle based on our finding that skeletal muscle CRP was elevated in the SO group *vs.* both the young and AO subjects (Figure 1C). Our results are in agreement with the InCHIANTI study, which demonstrated that high levels of pro-inflammatory cytokines (IL-6 and CRP) are significantly associated with poor physical performance and muscle strength in subjects 65 years and older [77]. Recently, Buford and colleagues (2010) have shown that nuclear factor kappa B (NF-κB) content, an evolutionarily conserved transcription factor involved in stimulating more than 150 genes involved in inflammation and protein turnover resulting in disuse-induced skeletal muscle atrophy, is significantly higher in the skeletal muscle of sedentary old *vs.* both young and active old groups [78]. In addition, recent studies have reported low-grade chronic inflammation in patients with osteoarthritis [79]–[82]. We believe that this systemic inflammation is secondary to physical inactivity due to osteoarthritis, and not a direct cause of pathology. We speculate that CRP-mediated chronic systemic inflammation may lead to long-term skeletal muscle damage, and thereby contribute directly to sarcopenia. Based on these observations and the self-reported activity pattern of our subject groups, we suggest that skeletal muscle CRP levels could potentially be used as an easily quantifiable diagnostic biomarker to assess lifestyle and activity levels of elderly individuals.

The mitochondrial theory of aging stipulates that an increase in free radicals oxidize macromolecules that would compromise the bioenergetics and functional capacity of the cell [28]. Mitochondria are not only the primary source of cellular metabolic energy production, but are also the major site of ROS production [41], [83]. It has been demonstrated that ROS, produced by the mitochondria, are maintained at a relatively high level within mitochondrial matrix [84]. Given the proximity of mtDNA and other mitochondrial proteins, it is possible that these molecules are at a greater risk of incurring oxidative insults and, in turn, leading to mitochondrial dysfunction [85]. Skeletal muscle from older adults and aged rodents have consistently shown an accumulation of somatic mtDNA mutations and large scale deletions, and a transcriptome “signature” indicative of mitochondrial dysfunction [32], [86]. Balagopal et al. (1997) reported a marked decrease in muscle mitochondrial protein synthesis rates in older adults [87]. Despite the reported detrimental effects of aging on mitochondrial redox status and mtDNA stability, the nature of the change in the mitochondrial ETC in human skeletal muscle is equivocal. Many studies have demonstrated a significant age-related reduction in mitochondrial ETC complex enzymes [34]–[37], while others failed to observe such changes [33], [38]–[41]. Barrientos and colleagues (1996) reported that after correcting for tobacco consumption and physical activity as confounding variables there was no apparent age-related decrease in mitochondrial oxidative capacity, suggesting that ETC function does not change as a result of ‘normal’ aging [38]. We first measured the protein content of PGC-1α, a master regulator of mitochondrial biogenesis that plays a crucial role in co-ordinating nuclear and mtDNA gene transcription [88]. The PGC-1α was not different between AO and young subjects (Figure 2A). Correspondingly, we observed similar levels of nuclear and mitochondrial DNA-encoded COX subunits as well as mitochondrial enzyme activity (COX and CS) in the AO compared to the young group (Figures 2A-C). On the other hand, SO adults had a significant reduction in PGC-1α and evidence of reduced mitochondrial biogenesis and mitochondrial COX activity in comparison with both the active old and young adults (Figures 2A-C). Since PGC-1α is proposed to be involved in co-ordinating mitochondrial biogenesis in response to exercise [89], [90], our findings suggest that physical inactivity may lead to lower PGC-1α and contribute to the subsequent reduction in mitochondrial capacity in the SO subjects. Surprisingly, we observed a significant positive correlation between complex IV activity and skeletal muscle strength across all the subjects in the study (Figure 2D). Indeed studies by Akien and colleagues have reported a direct link between mitochondrial aerobic capacity and skeletal muscle where mitochondrial abnormalities (decrease in mitochondrial activity and increase in mtDNA deletions) are positively associated with muscle fiber atrophy and splitting, and ragged red fibers in aged rats [27], [91]. Hence, this observation further supports our conclusion that an active lifestyle not only maintains muscle mitochondrial COX activity during aging, but also preserves skeletal muscle mass, an important predictor of mortality and aging associated co-morbidities [65].

Previous research has suggested that reduced PGC-1α mRNA expression is associated with lower mitochondrial function in aging [88], [92], [93]. Activation of PGC-1α has been identified as a potential therapeutic target for the treatment and prevention of many age-related declines in physiological function, including insulin resistance [94], inflammation [92], and muscle atrophy (i.e., sarcopenia) [95]. PGC-1α has also been implicated in the cellular antioxidant response [96], and our findings indicate that lower PGC-1α content may also contribute to the “redox crisis” in the SO subjects. In addition, Anderson and colleagues (2008) have shown that GSK-3β negatively regulates PGC-1α activity by targeting it to nuclear proteasomal degradation [97]. We observed a significant increase in GSK-3β activation (Figure 2A) in SO subject *vs.* both the young and AO groups which may also explain the reduction in PGC-1α content in SO. Our findings suggest that, at the protein level, a physically active lifestyle may maintain skeletal muscle PGC-1α expression, which likely contributes to improved mitochondrial capacity, antioxidant defence systems, and muscle performance. The reduction in PGC-1α, mitochondrial function, and antioxidant enzymes in the SO group provides indirect evidence that when aging is accompanied by a sedentary lifestyle, PGC-1α and the stimulus for mitochondrial biogenesis decline, potentially contributing to the reduction in mitochondrial function, increased susceptibility to oxidative stress, and reduced muscular strength.

Since we observed a dysfunction in mitochondrial COX activity in the skeletal muscle of SO group, we next investigated mitochondrial Mn-SOD which constitutes the first line of defence against O^-.^_2_ radical formation as a result of electron leakage from mitochondrial ETC [45]. We observed a significant reduction in both Mn-SOD protein content and activity in the SO adults relative to both the AO and young adults (Figures 3A and 3B). Interestingly, Mn-SOD activity was preserved in the AO adults, despite a significant reduction in their Mn-SOD content compared to the young (Figure 3B). There was no difference in cytosolic Cu/Zn-SOD protein content and enzyme activity between the three groups (data not shown). Since aging is widely associated with an increase in O^-.^_2_ anion production via mitochondrial ETC in skeletal muscle, we suggest that the mitochondrial antioxidant enzyme milieu of recreationally active older adults provides an intra-organellar environment conducive to O^-.^_2_ dismutation via maintaining Mn-SOD activity similar to that found in young subjects. This is in agreement with our previous findings where we demonstrated an age-associated increase in Mn-SOD activity in recreationally active older adults compared to young subjects [33].

The disagreement observed between Mn-SOD protein content and its activity in the AO adults is intriguing and suggests that Mn-SOD activity may be modulated by post-translational modification [66]. Pathological increases in cellular NO levels due to an increase in iNOS expression or overactivation of eNOS and nNOS result in the formation of peroxynitrite (ONOO^-^) radical [67]. The ONOO^-^ radical can cause lipid peroxidation and DNA damage, and is considered to be a potent tyrosine-nitrating species [66]. Exposure of human recombinant Mn-SOD protein to peroxynitrite *in vitro* causes highly specific nitration of tyrosine-34 at the active site of the Mn-SOD enzyme, resulting in inhibition of its catalytic activity [50]. Inactivation of Mn-SOD activity leads to futile cycling of O^-.^_2_, resulting in an amplification of oxidative stress [69]. We hypothesized that physical inactivity-associated dysregulation in mitochondrial COX activity may result in aberrant formation of free radicals, i.e., O^-.^_2,_ OH^.^, ONOO^-^, etc., via activation of NOS content, thus producing pathological levels of NO and causing inactivation of Mn-SOD in the skeletal muscle of the frail old subjects. Indeed, we observed that the skeletal muscle of the SO had higher amounts of both the nNOS content and nitrotyrosine content of immunoprecipitated Mn-SOD than both young and AO adults (Figures 4A and 4B). This phenomenon appears to be involved in ischemia-reperfusion injury, chronic rejection of transplanted organs, inflammatory diseases, neurological disorders (including amyotrophic lateral sclerosis, Parkinson's disease, and multiple sclerosis), atherosclerosis, and viral infections [66], [67], [69]. Together, these observations suggest that Mn-SOD activity in the skeletal muscle is maintained with a physically active lifestyle, and that sedentary lifestyle-mediated mitochondrial ETC dysregulation makes the intracellular environment more conducive to pro-oxidant production, resulting in the inactivation of Mn-SOD, insufficient dismutation of O_2_^-^ radical and mitochondrial redox crisis.

The findings of this study highlight the complexity of coordinated regulation of mitochondrial bioenergetic efficiency and cellular redox homeostasis in skeletal muscle, and functional capacity of older adults. We believe that a physically active lifestyle promotes redundancy and adaptations throughout the aging process via activation of antioxidant defence and repair pathways that allow the cell to maintain energy homeostasis despite the accumulation of abnormalities (somatic mtDNA mutations and deletions, and DNA, protein and lipid oxidation). Conversely, a sedentary lifestyle during aging is negatively correlated with these adaptations mainly due to the dysregulation of redox signaling and reduced mitochondrial function that renders the intracellular environment prone to pro-oxidant production and an aberrant redox homeostasis. Since a physically active lifestyle is suggested to have therapeutic potential against osteoarthritis, cancer, atherosclerosis, obesity, type II diabetes, sarcopenia, metabolic syndrome and associated co-morbidities, it is intriguing to speculate that maintenance of the mitochondrial bioenergetic capacity via regular exercise training may combat these pathologies.
